# Co-design of resources to support researchers to develop accessible and inclusive patient information leaflets for randomised controlled trials: the UK-based MAPLE project

**DOI:** 10.1186/s13063-026-09836-8

**Published:** 2026-06-09

**Authors:** Vikki Wylde, Emma Johnson, Kirsty Roberts, Sharon Brennan, Tessa Fulton, Naira Abdullahi, Catherine Jameson

**Affiliations:** 1 Bristol Medical School, University of Bristol, Bristol, UK; 2 National Institute for Health and Care Research Bristol Biomedical Research Centre, University Hospitals Bristol and Weston NHS Foundation Trust and University of Bristol, Bristol, UK; 3 National Voices, London, UK; 4 Member of the Public, Bristol, UK

**Keywords:** Research inclusion, Research participation, Literacy, Clinical trials, Accessible patient information leaflets

## Abstract

**Background:**

Low English language literacy and diverse communication needs are widespread in the UK. Provision of lengthy and complex patient information leaflets (PILs) for clinical trials can impose a barrier to research participation. Inclusive research design incorporates providing clear and accessible written information, often in a layered approach. This project aimed to co-design resources to support researchers to create visual, inclusive, and accessible PILs for randomised controlled trials (RCTs).

**Methods:**

Nineteen public and community involvement and engagement co-design workshops were held with nine groups, with a total of 150 attendees. Workshops were held with communities, patients, and charity representatives that are often marginalised in health research, including representation from racially marginalised communities, adults with learning disabilities and/or autism, adults with physical and mental health conditions, and the LGBTQIA+ community. A prototype modifiable accessible PIL was developed by the research team, designed using previously co-developed recommendations. During the first round of 10 workshops, a prototype accessible PIL was reviewed and discussed, and changes made to the phrasing, order, format, and layout. Additional feedback informed the development of guidance to researchers on the use of the accessible PIL. Suggestions for images for each section of the accessible PIL were collated and corresponding images sourced from online platforms. During the second round of nine co-design workshops, images were discussed and selected for inclusion, alongside recommendations for further edits and refinements to the accessible PIL and guidance to researchers.

**Results:**

The co-design process resulted in substantial changes to the prototype accessible PIL. A suite of resources were developed, including an adaptable accessible PIL that can be downloaded and modified offline for each RCT, a selection of images from which researchers can choose images to purchase from platforms to include in their accessible PIL, and additional guidance on the use and modification of the accessible PIL. The resources are available at https://maple.bristol.ac.uk/.

**Conclusion:**

These co-designed practical resources aim to support researchers to create more accessible and inclusive written information to reduce English language literacy as a barrier to participation in RCTs, as part of a layered approach to information provision.

**Supplementary Information:**

The online version contains supplementary material available at 10.1186/s13063-026-09836-8.

## Background

Inclusive research design is fundamental to improving population health and reducing inequalities in healthcare [1]. Clinical trial populations are often not representative of the wider patient population, with under-representation of people from marginalised communities [2, 3]. There is no single definition of underrepresented, underserved, or marginalised populations in clinical trials, as this is context-specific, but examples of commonly marginalised groups include people from minoritised ethnic backgrounds, people from disadvantaged socioeconomic backgrounds, people with learning disabilities, older people, and the LGBTQIA+ community [4]. Barriers to participation in clinical trials are multifaceted and complex, and can relate to language and communication, mistrust, access, attitudes and beliefs, eligibility criteria, lack of knowledge around clinical trials, and logistical and practical issues [5]. The provision of overly complex information is a barrier to accessible and inclusive clinical trials, particularly for people with lower literacy levels [4, 5]. Over 7 million people in the United Kingdom (UK) are estimated to have low English language literacy levels [6]. This can impact on health literacy and the ability to understand, appraise, and use written health information to make decisions about health. Low health literacy intersects with lower level of education, lower socioeconomic status, physical disability, and an ethnic minority background [7–9]. Patients approached about participation in a randomised controlled trial (RCT) are often provided with all the written information in a single patient information leaflet (PIL), that is typically long, dense, and has poor readability [10–15]. Through doing this, the research community is imposing a barrier to research participation, potentially preventing people with low English language literacy from participating by providing inaccessible information. A culture shift in the way information about RCTs is provided to patients is warranted to mitigate this barrier. The use of shorter and more accessible documents explaining what is important to patients in addition to the main PIL is recommended [16–18]. In the UK, there is mandatory information that must be provided to potential participants, for example on GDPR and data protection, to ensure they can make an informed decision about participation [16]. To balance these requirements with providing accessible information, giving information in stages, such as in a ‘layered approach’ [16], allows time for building on knowledge and understanding. There is a growing body of research providing guidance on how to provide accessible information in full PILs, such as describing randomisation, placebos, and benefits and harms [19–22]. However, practical resources to support researchers to create inclusive and accessible PILs for RCTs are lacking.

In phase 1 of this project, we worked in collaboration with people from racially marginalised communities, adults with a learning disability, patients, and charity representatives to develop recommendations for developing accessible PILs for RCTs [23]. This comprises a framework of 74 recommendations covering topics of formatting, information, presentation, writing style, content, and accessibility. Building on this framework of recommendations, the aim of this work was to co-design online resources to support researchers to create visual, inclusive, and accessible PILs for RCTs. Specific objectives were to develop an accessible modifiable PIL that could be downloaded and adapted offline for each RCT, identify a selection of images from which researchers could choose images to purchase from platforms to include in each section of their accessible PIL, and develop guidance on the use and modification of the accessible PIL.

## Methods

The MAPLE (making trials more accessible through better patient information leaflets) project was conducted as public and community involvement and engagement (PCIE) activities, working with communities, patients, and charities to co-design resources, and therefore institutional ethics approval was not required. The work was a partnership between the University of Bristol and National Voices, a coalition of health and social care charities in England.

### Development of the prototype accessible PIL

To facilitate workshop discussions, we used our published co-developed recommendations for accessible PILs [23] as a framework to develop content and layout for the preliminary prototype of the accessible PIL. For the content development, topic headings from the published recommendations were used to structure the content into sections. The sections developed for the initial prototype accessible PIL are provided in Supplementary material 2 (this prototype was subsequently updated through the iterative co-design process). One researcher (VW) then mapped the content of 10 example accessible PILs (shared with the research team during the previous phase of this project [23]) into each section heading. Content creation was supplemented by using online resources (e.g. NIHR Be part of research) to supplement examples from accessible PILs. Recommendations relating to formatting, information presentation, and writing style were incorporated into the design of the prototype accessible PIL. The academic team (VW, CJ, EJ, KR) then reviewed the mapped content to develop the first draft of section headings and text. Each PIL section was categorised as:

- Standardised: standardised content that can be used for any RCT
- Standardised with modifiable components: standardised content with modifiable components that need to be written by the research team
- Trial-specific: content needs to be written by the research team

An initial prototype accessible PIL was developed, alongside separate documents of text examples for each section for discussion in the first round of workshops (see Supplementary material 1 for an example). For trial-specific sections, example text was developed based on an ongoing trial evaluating radiofrequency denervation for chronic low back pain [24]. The prototype modifiable accessible PIL was populated with draft text to allow discussion of the layout and format (see Supplementary material 2). The recommendations from the first phase of the project were that each section should be accompanied by an image that helped to explain the text [23]. No images were included in the initial prototype accessible PIL that was shared during the first round of workshops to allow suggestions for images to arise organically through discussion.

### Format of workshops

The project involved 19 PCIE workshops with a total of 150 attendees over a 6-month period to co-develop resources to support researchers to develop accessible PILs for RCTs. Workshops were held with communities, patients, and charity representatives that are often underserved in health research, including representation from racially marginalised communities, adults with learning disabilities and/or autism, adults with physical and mental health conditions, and the LGBTQIA+ community. Two workshops were conducted with nine different groups, with one community group having an additional workshop as the first workshop was conducted separately for men and women. Although we aimed for the same people to attend both workshops, for some groups this was not possible due to people’s availability. We worked with groups with which we held existing and trusted relationships, formed new links with existing groups, and created new groups to expand diversity of voice within the project, using the collaborative principles learned through previous work [25]. A description of the groups and attendees at each workshop is provided in Table 1. Each workshop was 1–3 h in duration depending on the preferences, availability, or usual practice of the groups and facilitated by one or more community leader/staff facilitator and at least one academic member of the team. Detailed notes were taken live during the meetings or from an audio-recording of the meeting, depending on each group’s preferred way of working. Interpretation was provided by community members/leaders for three of the groups and a multilingual community health ambassador was present for a fourth group, although interpretation was not required. We provided context for the interpreters ahead of meetings, ensured that we were realistic about the topics to be discussed to allow up to double the time to discuss with interpretation, and paused often to allow ease of interpretation. We encouraged discussion in people’s preferred language, with the interpretation provided for the researcher. Payment for interpretation and/or facilitation was included in the budget. Further individual feedback was obtained from some attendees after the workshops where sufficient feedback had not been achieved in workshops due to the nature of group discussions and time constraints, where specific group representatives were absent from a workshop, or where we required greater input from a specific marginalised group.

**Table 1:** Summary of the groups and workshops

Group name	Group description	Workshop 1	Workshop 2
Chinese Community Wellbeing Society	Organisation to support the health and wellbeing of the Chinese speaking community in Bristol and across the southwest of England	Attendees: 8 group members Location: Vassall Centre, Fishponds, Bristol	Attendees: 8 group members Location: Vassall Centre, Fishponds, Bristol
Dhek Bhal	Charitable organisation that provides support and care for the older South Asian community in Bristol	Women’s group attendees: 15 group members Location: Wellspring Settlement, Barton Hill, Bristol	Attendees: 6 group members Location: Wellspring Settlement, Barton Hill, Bristol
		Men’s group attendees: 6 group members Location: Wellspring Settlement, Barton Hill, Bristol	
Eastern European community	Group convened for the project, supported by Caafi Health’s and Bristol Health Partners health ambassador scheme	Attendees: 7 group members Location: virtual meeting	Attendees: 8 group members Location: virtual meeting
My Friday Coffee Morning	A group for local residents to meet and discuss relevant social and health issues, with membership being majority women of Black, African, and Caribbean heritages	Attendees: 15 group members Location: Wellspring Settlement, Barton Hill, Bristol	Attendees: 8 group members Location: Wellspring Settlement, Barton Hill, Bristol
National Voices	Charity representatives from TransActual, OUTpatients, Learning Disability England, Roma Support group, and two lived experience partners	Attendees: 4 charity representatives and 2 lived experience partners Location: National Voices offices, London	Attendees: 3 charity representatives and 2 lived experience partners Location: virtual meeting
Patient Experience Partnership in Research (PEP-R)	PPI group at the University of Bristol comprising seven patients who had, or are having, treatment for musculoskeletal health conditions	Attendees: 6 group members Location: community venue, Southmead, Bristol	Attendees: 7 group members Location: community venue, Southmead, Bristol
Research abilities	PPI group of adults with learning disabilities in the Northeast of England, set up by the Lawnmowers Independent Theatre Company	Attendees: 9 group members Location: virtual meeting	Attendees: 10 group members Location: The Lawnmowers Arts Centre, Pelaw, Gateshead
The Brandon Adventurers	A group of autistic people and people with learning disabilities from across the South of England, supported by the charity Brandon Trust	Attendees: 8 group members Location: Brandon Head Office, Bristol	Attendees: 6 group members Location: virtual meeting
The Misfits Theatre Company	A theatre and social group led by people with learning difficulties	Attendees: 5 group members Location: St Pauls Learning Centre, Bristol	Attendees: 7 group members Location: St Pauls Learning Centre, Bristol

### Workshop 1

The first round of workshops began with an introduction to the purpose and aims of the project, followed by a facilitated discussion. The prototype accessible PIL was shared, and whilst the format of the discussion was tailored to the preferred ways of working for each group, we held discussion around how the group members prefer to receive information, barriers to taking part, initial thoughts on how they would feel if they received the PIL, and wider accessibility issues. The order of the sections, formatting, layout, and volume of information were discussed. The different text options for sections of the accessible PIL were read aloud and preferences for phrasing were discussed. For each PIL section reviewed, workshop attendees were asked to give suggestions for images that could be used to convey the key message of the section. Sections of the accessible PIL were reviewed by different groups, with all sections having input from at least two groups.

Feedback from the 10 workshops was collated into one document and reviewed by a researcher (VW). For each feedback point, a recommended action was developed. These recommended actions were then reviewed and discussed by all members of the research team to ensure they reflected the workshop discussions. The majority of recommended actions were implemented and resulted in changes being made to the text, order, formatting, and layout of the accessible PIL. Some recommended actions were not implemented, for example where the suggestion of one group was different to the majority of other groups. The research team developed a ‘guidance to researchers’ document which used feedback from the workshops to provide further advice and guidance in adapting the PIL for different RCTs, including explanations of why each of the sections is important, and additional accessibility guidance.

All image suggestions were imported to a word document and a researcher (VW) and a paid member of one of the community groups (co-author NA) searched online platforms to identify images that reflected each suggestion, with a focus on diverse representation.

The revised accessible PIL was also discussed during the second workshop and further feedback sought. Another round of analysis of the feedback was undertaken to develop recommended actions, and further refinements were made and implemented.

### Workshop 2

The second round of workshops focused on the images that should be included as part of the online resources. Images that were sourced for each suggestion from the first workshop were reviewed and workshop members provided feedback, with a focus on whether the image was useful for conveying the key message of the section, was clear and easy to understand, and was inclusive (i.e. to a diversity of abilities, ethnicities, gender identities, etc.). A total of 109 images were reviewed during the second round of workshops. Images for each PIL section were reviewed by at least four groups. Images were included in the resources if there was general consensus across the groups. Images that were considered unclear or inappropriate by the majority of groups were excluded. Images where opinions differed between or within groups were reviewed in the workshop with National Voices members, and a final decision was made. Recommendations for new images arose during the second workshop; images for these suggestions were sourced and feedback sought from members of the group who made the suggestion to decide if the image should be included.

### Development of the website

To make the resources available and accessible for use by trial teams, a website was created by the University of Bristol Research IT team using Bristol Blogs, in consultation with the academic team and an external User Experience consultant. To inform the design of the website, the User Experience consultant held discussions with a range of target, trial staff ‘end-users’, to ensure website functionality fitted with need. The final website design was reviewed by the National Voices group.

## Results

The resources are available at https://maple.bristol.ac.uk/. The modifiable accessible PIL is free to use for non-commercial research and can be downloaded from the University of Bristol’s express licensing portal and customised offline to be tailored for individual RCTs. Guidance is provided on the website to support researchers in adapting the accessible PIL for their trial. Example images and/or guidance on selecting images is provided for each section of the PIL. Each image has a link to the host platform where the image can be purchased for use. Full guidance on how to modify and use the accessible PIL is provided on the website; below we provide an overview of each section of the PIL, highlighting key examples of how the co-design process informed development of the resources.

### Study title

A recommendation that arose during the co-design process was that the study title on the accessible PIL should contain no medical jargon, as this could put people off reading the accessible PIL. It was also considered important that the title included ‘Information leaflet for a clinical trial’ rather than ‘Information leaflet’ to ensure it was clear that this is information for an RCT, rather than information about the intervention(s), patient care, or condition described.

### ‘What is this clinical trial about?’ section

Discussions of this section highlighted the need to avoid using medical jargon as much as possible. However, it was acknowledged that some medical terminology may be unavoidable, e.g. the name of the intervention. Therefore, providing an explanation of the term prior to use, followed by the medical term in bold was considered an acceptable approach to address this issue. To accompany this change, an explanation that medical terms are in bold was added at the beginning of the accessible PIL. It was considered important for researchers to make the nature of the intervention clear, to include information about what is already known about the intervention(s) being tested and why the RCT is needed. If the trial is evaluating a novel intervention, it was recommended to include an explanation of what research has already been done prior to this trial, with a focus on safety.

### ‘Why have you asked me?’ section

This section was considered important to clearly explain why people had been asked to take part in the RCT. It was important that this is situated early in the accessible PIL so that people can determine whether it is of interest and relevance to them and if they are eligible from reading the first page. It was recommended that eligibility criteria should be written clearly and in plain language, with each eligibility criterion described in a bullet point list. If the eligibility criteria list is long, then key eligibility criteria should be provided.

### ‘Do I have to take part?’ section

In this section, it was considered important that emphasis is placed on the voluntary nature of participation, that it is the patient’s decision whether or not to participate, encourage discussion with other people, let patients know they can ask the research team questions to help with decision-making, and provide reassurance that patients will still receive their usual medical care if they decide not to participate.

### ‘Are clinical trials safe?’ section

During the co-design process, fear and mistrust arose as important barriers to research participation, and therefore the key message in this section is that safety and participant wellbeing are central in an RCT. This section describes the regulation and oversight of RCTs in plain language. Groups discussed how they and/or their communities may not be familiar with the term ‘ethics committee’ and therefore this term was removed. It was agreed that it was sufficient to explain that all RCTs are reviewed by experts who protect the rights, safety, and wellbeing of participants. Transparency in reporting concerns or complaints was important, and therefore contact details for an independent organisation, such as the Patient Advice and Liaison Service, were added.

### ‘Which treatment will I get?’ section

This section focuses on providing a clear explanation of the randomisation process and treatment groups. Discussions highlighted that the following points cover the key information that is important for patients to understand when deciding if they would like to participate in an RCT: how many trial arms there are, how treatment allocation will be conducted and the reasons why random allocation is important, key differences between the treatments being tested, and whether patients will be informed of their treatment allocation. During the co-design process, a plain language description of these core design features was developed. For describing the randomisation process, it was highlighted as particularly important to avoid descriptions such as tossing a coin, rolling dice, or a computer deciding as these descriptions may make people feel that their health is being trivialised.

For describing the treatment groups, subheadings were added to improve readability. It was considered important that the treatments and associated risks/side effects are clearly described and that the amount of information is proportionate to the intervention; RCTs of innovative procedures, high-risk RCTs or those with a complex design, will require a longer section explaining the procedures and risks. During the development of this accessible PIL, discussions highlighted the beliefs held in popular culture that people associate with the term ‘placebo’, i.e. the ‘placebo effect’, and that patients receiving a placebo treatment may be being ‘tricked’ into a response. It was therefore agreed to change the term to ‘inactive treatment’, being described as a treatment that looks the same as and is given the same way as the active treatment but has no known effects.

### ‘What will I need to do?’ section

The co-design process highlighted that the core information to present in this section relates to the study processes, including what will happen at each stage of the study and the time commitment over the life of the study, to support informed decision-making. Processes for follow-up and data collection, including the different ways that data will be collected, the duration of follow-up, and additional support available with providing outcome data, were considered important to describe in this section. During workshop discussions, some attendees highlighted that for some people from minoritised groups, disclosing demographic information can make them feel vulnerable and therefore text was added to explain that it is people’s choice whether they provide this.

### ‘What will happen to my information?’ section

The text for this section in the prototype accessible PIL was considered by the groups to convey the key information that they would want to know to make an informed decision about participation: that personal information will be kept confidential and stored securely and that individual participants will not be identifiable in any anonymised data that is shared. Whilst no changes were made to the text in the accessible PIL, discussions with one group highlighted that the possibility of confidentiality being breached for safeguarding concerns may impact negatively on building trust between the patient and the trial team. This was added to the guidance to researchers, explaining that further elaboration on this may be required during discussions between trial site staff and patients, such as explaining what would trigger a breach of confidentiality, which services would be contacted, and what information would be shared.

### ‘What if I do not want to carry on with the clinical trial?’ section

People involved in the co-design process wanted clear information that patients could stop taking part in the RCT at any time, without giving a reason. Information on how patients could withdraw from the RCT was considered important and this was added to the accessible PIL.

### ‘Thank you’ section

One of the recommendations from our previous work was that it is important to let patients know that their time and contribution to health research is valued and appreciated. Through the co-design process, it became apparent that this does not necessarily need to be through payment for participation, but that patients should be told about the trial findings and given a timeframe for this. It is of particular importance to ensure sharing of results as part of building trust with minoritised communities, through improved transparency and clarity within research culture. Informing participants of the trial findings was considered key to build trust and relationships between patients and research teams. Discussions also emphasised the importance of being clear that any payment for participation will be offered rather than given as some people may not want to receive payment (for example due to concerns about impact on benefit payments); this was added to the guidance to researchers.

### ‘What next?’ section

This accessible PIL is designed to be part of a layered approach to information and therefore signposting to accessible sources of further information for people who are interested and want to find out more about the study was considered the key information to include in this section. People wanted clear information about next steps and how to express an interest in taking part in the trial. Through the co-design process, this section was changed to focus on explaining how further information would be provided, with timescales. Additionally, it was clarified that patients could contact the research team to request information in their language if required.

### ‘More information about clinical trials’ section

‘Clinical trial’ is a research term that may not be accessible to everyone, so it was considered important to include a short and simple explanation of RCTs, alongside a section to help people understand the value of participation in RCTs and how their contribution to medical research is vital to help others. The accessible PIL includes two sections—‘What is a clinical trial?’ and ‘Why is taking part in clinical trials important?’ During the co-design process, it was highlighted that some patients may like more general information about RCTs, and therefore the accessible PIL was amended to encourage patients to contact the research team for more information.

### Images

The key criteria for selecting images were that they were clear, unambiguous, help to communicate the main points of the corresponding text, represent diversity, and are authentic and inclusive. It was acknowledged that it can be challenging to represent a wide range of diversity in a single image and therefore it was recommended that images used across the whole accessible PIL show diverse and intersectional representation. Members of the National Voices workshop emphasised that not all aspects of diversity are visible, and it is important to work with people with lived experience to understand how diversity can best be represented and whether photographs or illustration is the more appropriate format. Ensuring consistency in image type throughout the accessible PIL, such as all photos or all illustrations, was considered important.

### Order, structure, and formatting

The order of the accessible PIL changed substantially during the co-design process. Initially, the general information about RCTs featured on the first page. However, these were moved to the end of the accessible PIL and the sections on ‘what is this clinical trial about?’ and ‘why have you asked me?’ were moved to the first page so that people could determine from reading the first page whether the RCT was of interest and relevance to them. Another important major modification was the removal of sections describing the advantages and disadvantages of participation. The views of the people involved in the co-design process were that having these sections could introduce researcher bias as the advantages and disadvantages that may not be the same for all participants. They recommended that by clearly describing the study processes and the treatment options, patients will have the information they need to decide what the advantages and disadvantages of participation are for them and whether they would like to participate.

The co-design process led to the addition of further information at the start of the PIL. A greeting in the main languages spoken by the groups we worked with was included to emphasise that the RCT is for everyone. A sentence explaining who people need to contact if they need to speak in their language, with contact details, was added so that people whose first language is not English do not need to read the whole PIL to find the contact details at the end.

It was important to the people involved in the co-design process to keep the PIL as short as possible, with a maximum of 4–5 sides of paper. A minimum font size of 15 was recommended, whilst acknowledging that some people will require larger font size or braille. The preferred font colour was black, with headings in a different colour to distinguish these from the section text. Provision of the modifiable accessible PIL in a plain template was considered most appropriate, as this would allow trial teams to personalise the accessible PIL to make a unique and visually appealing source of information for their RCT.

### Further guidance

Consideration should be given to the need for language services, either interpretation or translation, and to include the costings in trial budget planning [26]. A conversation with someone whose first language is not that spoken by the recruiter and requires interpretation may take longer; this should be considered at trial planning stage and sites supported to spend extra time holding these conversations. People may wish to receive translated written materials. However, feedback from certain groups was that in some communities, it is common that people who speak a language do not read it, so it should not be assumed that translated materials are the solution.

The groups raised the need to consider how to reach everyone who may be eligible for the trial, taking steps to advertise the trial to marginalised communities. It was recommended that researchers establish and utilise links with charities, community groups, and grassroots organisations to promote the trial to their members, via public involvement and engagement specialists, if available. Researchers should further consider the method of initial contact. For example, an unsolicited postal invitation is less likely to gain traction than a personal invitation from a trusted healthcare professional.

Customised versions of the accessible PIL may be appropriate, adjusted to the needs of specific communities, for example those who are at increased risk of the condition being studied.

## Discussion

This project was conducted in partnership with minoritised communities, patients, and charity representatives who may be marginalised by current practices in healthcare research, for example by provision of overly complex information, by language, or by lack of diverse representation in study materials. We developed a suite of practical resources to support researchers to create accessible PILs for RCTs. This article provides an overview of the methods used and the impact of the co-design process on the resources developed. The project builds on previous work that co-produced a comprehensive framework of recommendations to guide researchers in the development of accessible PILs for RCTs [23]. In acknowledgement that creation of guidance may not be sufficient to support change, these novel and practical resources were developed to promote implementation of the recommendations.

We have described the need for a culture shift in the way that information is provided to potential trial participants. We also advocate for a culture shift in language, for example, over the lifetime of this project, we have changed the focus from describing people’s low literacy levels as the barrier to inclusion, to the barrier being the provision of overly complex and exclusive information. This shift places the barrier within research practice, and therefore the responsibility on researchers to take steps to make trials accessible to all; we must be aware that language in common use within research may perpetuate bias and be open to acknowledge, challenge, and change. Provision of accessible written information about RCTs is important to reduce the exclusion of people with lower English language literacy and facilitate recruitment of more diverse and representative samples in RCTs, thereby improving equity and generating trial findings that are generalisable to the wider patient population. The accessible PIL also has the potential to introduce efficiencies in the research process, negating the need for research teams to design the full content for accessible PILs for every RCT. This may also lessen the burden on minoritised communities of researchers seeking advice about writing generic sections for accessible PILs. Availability of these resources does not remove the need for PCIE in RCTs but introduces scope for PCIE to be more focused, nuanced, meaningful, and trial-specific.

During early discussions with communities, we heard that example PILs that we provided for discussion felt as if the information provided was from the point of view of researchers ‘covering themselves’ rather than through the participant lens. The aim of this accessible PIL is to first provide patients with a concise document containing the information that they want to know about an RCT, rather than the information that research community thinks they should be told, to help patients make an informed decision about whether they would like to receive further information, as recommended in guidance on designing inclusive RCTs [17]. Researchers perceive regulatory authorities as a barrier to use of accessible PILs, due to the need to meet regulatory and legal requirements [27]. However, the Health Research Authority recommends a layered approach to information provision [28], to ensure that clinical research is accessible and communicated well to people in line with the principles and hallmarks of people-centred clinical research [29]. In the UK, there are legal requirements to provide information to potential participants, for example on GDPR and data protection. This can be incorporated within a layered approach, providing the information that is important to patients first, before them providing more detailed information to those who are interested to ensure the information provided meets clinical trial regulations. However, to continue to mitigate against excluding people with lower English language literacy, the accessibility and format of further information also need to be considered. To optimise inclusivity, guidance recommends a greater focus on patient-centredness and community involvement in the research process, including supplementing written materials with other formats, such as multimedia, videos, and illustrations [4, 26]. This was echoed in group discussions. Initial contact methods should also be considered and trust is key; creating, maintaining, and utilising links with community and grassroots organisations to share or promote the trial may help uptake from marginalised communities, and a face-to-face discussion with a healthcare provider may be preferred over other impersonal invitations to participate in a trial. Language services must be considered to provide interpretation or translated material where needed. To feel included and valued, people need to see themselves reflected; therefore, trial materials must include diverse and intersectional visual representation of minoritised communities.

It is important to consider the strengths and limitations of this work within a broader context. The project was conducted as a partnership between an academic organisation and a coalition of health and social care charities, which facilitated involvement of people with relevant lived experience from a wide range of backgrounds relevant to this project. The ability to hold these workshops built on many years of work, both in the academic and charity sector, to build trust and relationships with people who have been disadvantaged and minoritised by healthcare and health research. For example, the academic team has been working closely with a number of the community groups for over the last 4 years, to build trusted relationships and to understand how to conduct inclusive involvement in health research [23, 25, 30]. These principles were used to create new relationships to widen representation in this project. Whilst the project involved representation from racially marginalised communities, adults with learning disabilities and/or autism, adults with physical and mental health conditions, and the LGBTQIA+ community, there may be other lived experiences relevant to this project that were not represented in the workshops. We did not collect data on protected characteristics on workshop attendees and therefore we cannot comment further on this; instead, we focused on involving people based on the community group or organisation that they were part of. One key factor associated with low literacy is lower socioeconomic status and we did not specifically approach groups based on this. It is also acknowledged that this project was limited in scope and only addresses one component of inclusive research design. The project was focused on the provision of accessible written information in English for RCTs. Further work is needed on providing translated materials, information in other formats, and developing modifiable accessible PILs for other research designs; we see this project as providing the foundation of accessibility and inclusion upon which to build this work. Finally, although the accessible PIL and guidance was developed with consideration for different trial contexts, trial teams are encouraged to work in collaboration with patients and the public to ensure the accessible PIL contains all the relevant information to each particular trial design.

In conclusion, these co-designed practical resources aim to support researchers to create more accessible information to reduce the exclusion of those with lower English language literacy in RCTs and to increase diversity through more inclusive representation. These resources form part of a layered approach to information provision. Future work is planned to evaluate uptake, use and impact of the resources, and identify areas for improvement.

## Supplementary Information

Supplementary Material 1.

Supplementary Material 2.

Supplementary Material 3.

## Data Availability

The datasets generated and/or analysed during the current study are not publicly available as they comprise notes taken during meetings with community groups, patients, and charity representatives and we do not have permission from group members to share these notes.

## Abbreviations

MAPLE study: Making trials more Accessible through better Patient information Leaflets
PCIE: Public and community involvement and engagement
PPI: Patient and public involvement
PEP-R group: Patient Experience Partnership in Research
PIL: Patient information leaflet
RCT: Randomised controlled trial
HRA: Health Research Authority
