# Supplementary material for: Co-design of resources to support researchers to develop accessible and inclusive patient information leaflets for randomised controlled trials: the UK-based MAPLE project
Source: Trials. 2026 Jun 9;27:528. doi: 10.1186/s13063-026-09836-8 (PMC13471343; doi:10.1186/s13063-026-09836-8)
Supplement: Supplementary file 1 [file 13063_2026_9836_MOESM1_ESM.docx]

**2. Invitation to take part in a study**

**Options for sentence 1 - Ask if people would like to take part:**

- We are inviting you to take part in a study called a ‘clinical trial’
- You are invited to join a study called a ‘clinical trial’
- We are asking if you would like to take part in a study called a ‘clinical trial’

**Options for sentence 2 - Emphasise that taking part is their choice:**

- You can choose if you would like to take part or not.
- It is up to you if you want to take part.
- It is your choice if you want to join in the study.
- You do not have to join.
- It is up to you to decide whether or not to take part.

**Options for sentence 3 - Encourage discussion with other people**

- Please feel free to talk to other people about the study.
- You can talk to other people to help you decide.
- If you want to, please discuss with other people before you decide whether or not to take part.

**Options for sentence 4 – Encourage questions to the research team**

- You can ask us any questions before deciding.
- Please ask us if anything is not clear.

**Options for sentence 5: Medical care**

- There will be no impact on your medical care if you choose not to take part.
- Your medical care will not be affected if you decide not to take part.
- If you decide not to take part, you will still get the same healthcare.
