# Supplementary material for: Co-design of resources to support researchers to develop accessible and inclusive patient information leaflets for randomised controlled trials: the UK-based MAPLE project
Source: Trials. 2026 Jun 9;27:528. doi: 10.1186/s13063-026-09836-8 (PMC13471343; doi:10.1186/s13063-026-09836-8)
Supplement: Supplementary file 2 [file 13063_2026_9836_MOESM3_ESM.docx]

| **Section and topic** | **Item** | **Reported on page No** |
| --- | --- | --- |
| 1: Aim | Report the aim of PPI in the study | 5 |
| 2: Methods | Provide a clear description of the methods used for PPI in the study | 5-10 |
| 3: Study results | Outcomes—Report the results of PPI in the study, including both positive and negative outcomes | 10-18 |
| 4: Discussion and conclusions | Outcomes—Comment on the extent to which PPI influenced the study overall. Describe positive and negative effects | 18-20 |
| 5: Reflections/critical perspective | Comment critically on the study, reflecting on the things that went well and those that did not, so others can learn from this experience | 21 |

GRIPP-2 Short-form reporting checklist
